# Multi-task localization of the hemidiaphragms and lung segmentation in portable chest X-ray images of COVID-19 patients

**DOI:** 10.1177/20552076231225853

**Published:** 2024-02-01

**Authors:** Daniel I Morís, Joaquim de Moura, Shahab Aslani, Joseph Jacob, Jorge Novo, Marcos Ortega

**Affiliations:** 1 Centro de Investigación CITIC, 16737 Universidade da Coruña, A Coruña, Spain; 2 Grupo VARPA, Instituto de Investigación Biomédica de A Coruña (INIBIC), 16737 Universidade da Coruña, A Coruña, Spain; 3 Department of Computer Science, Centre for Medical Image Computing, 4919 University College London, UK; 4 Satsuma Lab, Centre for Medical Image Computing, 4919 University College London, UK

**Keywords:** COVID-19, chest X-ray, heatmap regression, lung segmentation, hemidiaphragm localization

## Abstract

**Background:**

The COVID-19 can cause long-term symptoms in the patients after they overcome the disease. Given that this disease mainly damages the respiratory system, these symptoms are often related with breathing problems that can be caused by an affected diaphragm. The diaphragmatic function can be assessed with imaging modalities like computerized tomography or chest X-ray. However, this process must be performed by expert clinicians with manual visual inspection. Moreover, during the pandemic, the clinicians were asked to prioritize the use of portable devices, preventing the risk of cross-contamination. Nevertheless, the captures of these devices are of a lower quality.

**Objectives:**

The automatic quantification of the diaphragmatic function can determine the damage of COVID-19 on each patient and assess their evolution during the recovery period, a task that could also be complemented with the lung segmentation.

**Methods:**

We propose a novel multi-task fully automatic methodology to simultaneously localize the position of the hemidiaphragms and to segment the lung boundaries with a convolutional architecture using portable chest X-ray images of COVID-19 patients. For that aim, the hemidiaphragms’ landmarks are located adapting the paradigm of heatmap regression.

**Results:**

The methodology is exhaustively validated with four analyses, achieving an 82.31% ± 2.78% of accuracy when localizing the hemidiaphragms’ landmarks and a Dice score of 0.9688 ± 0.0012 in lung segmentation.

**Conclusions:**

The results demonstrate that the model is able to perform both tasks simultaneously, being a helpful tool for clinicians despite the lower quality of the portable chest X-ray images.

## Introduction

The COVID-19, caused by the virus severe acute respiratory syndrome coronavirus 2, is a multi-organic infectious disease that, therefore, can affect many parts of the body, but that mainly affects the lungs and their surroundings.^1^ COVID-19 is an acute disease that can improve or worsen very quickly over time, where the patients can still experience symptoms once they test negative. Those symptoms can last for several days or weeks, as it happens with other pathologies such as the common flu, but they can also last indefinitely. When this happens, the patients are diagnosed with persistent post-COVID-19 syndrome (PPCS).^2^ This syndrome can severely affect the life quality of the patients in some cases. In particular, given that COVID-19 can affect the surroundings of the lungs, the diaphragm could be damaged, an important muscle involved in the breathing process.^3^ A dysfunctional diaphragm (i.e. weak or paralyzed diaphragm) can present several root causes apart from PPCS, such as stroke, nervous system diseases (for instance, multiple sclerosis or amyotrophic lateral sclerosis) or problems that affect the phrenic nerve.^4^ Given the great importance of the diaphragm in the breathing process, a dysfunction can cause shortness of breath, sleeping disorders and fatigue, among other symptoms. The diaphragm dysfunction has been thoroughly studied in other common pathologies such as chronic obstructive pulmonary disease, as a reference.^5–7^ This dysfunction can be assessed determining the gap between both sides of the diaphragm, often known as hemidiaphragms. This distance can be useful to quantify the breathing capacity of the patient in a given timepoint and to understand its evolution through time. This process can be performed with imaging modalities such as lung ultrasound (LU),^8^ chest X-ray^9^ or computerized tomography (CT).^10^ In particular, chest CT images provide a three-dimensional captures of the explored area with a great resolution and level of detail. However, this image modality is more expensive and difficult to perform. Given that the hardest peaks of the pandemic caused a saturation of the healthcare services, the preferred solution was chest X-ray imaging, as it is easier to manage when dealing with a great amount of patients in a small amount of time. In addition to these difficulties, during the pandemic it was necessary to prioritize the use of portable chest X-ray devices over fixed machinery.^11^ This prioritization was motivated by the fact that portable chest X-ray devices are easier to decontaminate, a critical element to prevent the risk of cross-contamination. Moreover, many critical patients required to remain in bed due to their condition, being unable to move to the radiology room. In this sense, the portable devices can be moved to and used where the patient is placed, an aspect that solves the previously mentioned issue. Despite all these advantages, the main issue of using this kind of devices is the low quality and level of detail of the captured images. In this context, the development of computer-aided diagnosis (CAD) systems can be studied as an option to help the clinicians quantify the diaphragmatic function of a given patient.

Apart from the assessment of the diaphragmatic function, the segmentation of several structures of interest in the lungs using imaging modalities as chest X-ray is also a relevant task. The main aim of these tasks is to automatically obtain the region of interest (ROI) of the image, removing information that could introduce noise to the CAD system. This can help to improve the performance of other tasks such as COVID-19 screening and classification in chest X-ray images, scope that has seen a great amount of contributions since the pandemic began (as reference,^12–19^). In this context, some works have been proposed. As reference, the work from Aslan^20^ proposes a method to diagnose COVID-19 in this kind of imaging modality using a DeepLabV3+ architecture for lung segmentation as a part of its pipeline. Other works use the U-Net architecture to perform the lung segmentation, as is the case of Rahman et al.^21^ and Vidal et al.^22^ In the latter case, the authors use a U-Net model that was pre-trained on a dataset of brain MRI images to segment the lungs in portable chest X-ray images. On the other hand, Alam et al.^23^ proposed a modified U-Net architecture that replaces skip connections with bidirectional convolutional long short-term memory modules to perform the lung segmentation task. In particular, related with the diaphragmatic function, the segmentation of the lungs can be helpful to determine the boundaries and the position of this structure. Therefore, to simultaneously perform the localization of the hemidiaphragms’ landmarks and the lung segmentation can help the model to do both tasks more accurately. In this sense, the multi-task learning is a framework commonly used in the state-of-the-art, that exploits the advantages of training with two or more simultaneous tasks^24^. Given the impact that the COVID-19 has made in the last years, many works have explored different problems proposing multi-task paradigms, using chest X-ray images. As reference, the work of Park et al.^25^ develops a methodology composed of a shared backbone based on a transformer encoder architecture and two different heads to perform the task of COVID-19 classification and the task of severity assessment simultaneously. In the case of Malhotra et al.^26^, the authors proposed a model called COMiT-Net. This model has a multi-task structure that simultaneously detects if an image presents COVID-19 affectation or not and shows the symptomatic regions with a semantic segmentation. Moreover, the application of multi-task learning has also been explored with datasets of CT images. As reference, the work of Polat^27^ proposes the use of DeepLabV3+ to segment COVID-19 lesions. The purpose of using multi-task is to simultaneously segment those lesions with several levels of detail, ranging from a binary segmentation (distinguishing between lesion and no-lesion) to a more detailed semantic segmentation (distinguishing between different types of lesions such as consolidation or pleural effusion, etc.).

The characterization of the position and the movement of the diaphragm is an important task to assess the diaphragmatic function. The contributions in this specific scope can be mainly found in LU or CT images, but there is also some literature in the field of chest X-ray. As reference, Heidari et al.^28^ proposed several preprocessing strategies to improve the performance of a convolutional neural network (CNN) trained to detect COVID-19 in chest X-ray. As part of these preprocessing strategies, the authors include a diaphragm removal using a plane threshold. Overall, it can be obtained that none of the works of the state-of-the-art have proposed a methodology to localize the hemidiaphragms’ landmarks with the potential to determine the gap between both structures of interest or related biomarkers. Related with this, it is remarkable that the localization of landmark points is a critical task in many computer vision problems, such is the case of face landmark detection.^29^ This also applies to many biomedical imaging problems and modalities.^30^ One straightforward approach for landmark detection using deep learning is an end-to-end paradigm that is fed with the input image and returns the coordinates of the detected points. However, this type of paradigms lose part of the strengths of the convolutional network architectures, as the local connectivity or the weight sharing. To exploit the full capability of this kind of architectures, many biomedical imaging works have presented the so-called heatmap regression as part of their pipeline.^31^ As reference, Silva et al.^32^ proposed an automatic pipeline composed of different steps to assess the severity of petum excavatum in CT images. The main target of this methodology is to perform several measures on relevant slices, given different landmarks. In particular, the authors consider the use of heatmap regression to detect those landmarks. In Kirnbauer et al.^33^, the authors develop a methodology to detect periapical lesions in cone-beam CT images. For this aim, it is necessary to obtain the coordinates of certain objects, such as the teeth. In this scenario, the heatmap regression method is used to predict the coordinates. Regarding the scope of retinographic imaging, several works have proposed the heatmap regression for tasks such as fovea localization or the localization of the center of the optic disc. As reference, this is the case of Meyer et al.^34^, Hervella et al.^35^, Al-Bander et al.,^36^ or Marin et al^37^.

To the best of our knowledge, none of the current state-of-the-art methods address the challenges of localizing the hemidiaphragms’ landmarks in portable chest X-ray images of COVID-19 patients. Portable chest X-ray imaging presents unique challenges compared to fixed imaging, including lower image quality and the potential for patient movement during the imaging process. Additionally, existing methods do not offer a solution for simultaneously localizing the hemidiaphragms’ landmarks and segmenting lungs in other pathologies.

To fill this gap in the literature, we propose a novel fully automatic deep learning methodology that employs a heatmap regression paradigm to simultaneously localize the hemidiaphragms’ landmarks and segment the lungs in chest X-ray images. The generator architecture of our method is based on a fully convolutional network that consists of an encoder and a decoder. The encoder extracts features from the input image, while the decoder generates the output heatmap for the localization of the hemidiaphragms’ landmarks and the binary mask for lung segmentation. We introduce an ensemble loss function that combines the dice loss for lung segmentation and the mean squared error (MSE) loss for localization of the hemidiaphragms’ landmarks to facilitate the learning of the generator.

To validate the feasibility and potential of our approach, we conduct an exhaustive study that includes four different analyses. Through these analyses, we demonstrate the effectiveness and potential of our proposed methodology in addressing the challenges of multi-task localization of the hemidiaphragms’ landmarks and the precise lung segmentation in portable chest X-ray images of COVID-19 patients.

- Analysis I: Ablation study to find the most appropriate value of saturation distance for the heatmap regression. This analysis explores the impact of saturation distance on the performance of the model, as this parameter is crucial in heatmap regression. The study involves training and testing the model with different saturation distances and comparing the results to determine the optimal value that produces the best localization of the hemidiaphragms’ landmarks. In this analysis, we also include a statistical test to support the given discussions.
- Analysis II: Study of the optimal balance between both tasks with regard to the training process. In this analysis, we investigate the best approach to balance the training process of the two tasks: hemidiaphragms’ landmarks localization and precise lung segmentation. The study aims to determine the optimal ratio of weights assigned to each task during the training process, which results in the best performance of the overall system.
- Analysis III: Comparison of the performance obtained by each task separately with the performance achieved when carrying out both tasks simultaneously. This analysis is important to demonstrate the added value of the proposed complete system, as the simultaneous execution of both tasks enables a more efficient and accurate localization of the hemidiaphragms’ landmarks and precise segmentation of the lungs.
- Analysis IV: Qualitative discussion of the outputs returned by the system. The goal is to determine the robustness of the model in various scenarios, such as the presence of abnormalities in the chest X-ray images, variations in patient positioning, and the impact of the use of portable chest X-ray images. This analysis also allows for the identification of potential areas for improvement and future work. Moreover, in this analysis, we have also included the study of the activation maps provided by the model using the GradCAM algorithm.

The rest of the article is structured as follows. Firstly, the ‘Materials and methods’ section describes the used dataset (‘CHUAC dataset’ subsection), the overall steps of the methodology (“Methodology” subsection) and the different details of the training process (“Network architecture and training details” subsection). After that, the “Results and discussion” section details the results obtained after the experimentation was performed, with their corresponding discussion. Finally, the “Conclusions” section discusses the main conclusions extracted from the work development and some possible lines of future works.

## Materials and methods

In this section, we detail the aspects of the used dataset in the “CHUAC Dataset” subsection and the used software and hardware resources in the “Software and hardware resources” subsection. Moreover, the description of the methodology is shown in the “Methodology” subsection and the details of the training process are described in the “Network architecture and training details” subsection, with special focus on those aspects that differentiate each task and the particular needs for the multi-task paradigm. Finally, the used evaluation metrics are explained in the “Evaluation metrics” subsection.

### CHUAC dataset

In this work, we have used a dataset of portable chest X-ray images provided by the Complexo Hospitalario Universtario de A Coruña (CHUAC), specifically designed for the purposes of this work. The dataset is exclusively composed of COVID-19 patients, making a total of 673 images, that were captured during the first peak of the pandemic in 2020. The dataset was manually labeled, including the manual segmentation of both lungs and the position of the 2 hemidiaphragms’ landmarks, having a total of 1346 labeled lungs. The images were obtained with 2 different portable machines, whose models are Agfa dr100E and Optima Rx200. Due to the previously-mentioned risk of cross-contamination, the captures were performed in isolated medical wings specifically intended to treat COVID-19 patients. The subjects were captured in supine position with an anterior-posterior projection. To do so, the device has a flexible arm with the X-ray tube, that can be placed over the patient. Then, a recorder plate placed under the patient is responsible for obtaining the capture. The resolution of the images ranges from 949×827 pixels to 1526×1910 pixels. It is remarkable the complexity of the studied scenario, given that the captures must be carefully filtered from pathological cases that can have affectation compatible with COVID-19 but caused by other diseases such as more common types of pneumonia. Moreover, the critical condition of some patients and the way the images are captured by the portable devices imply a great heterogeneity with the position of the subjects, in contrast with the fixed machinery where patients can be positioned more precisely.

**Figure 1.**
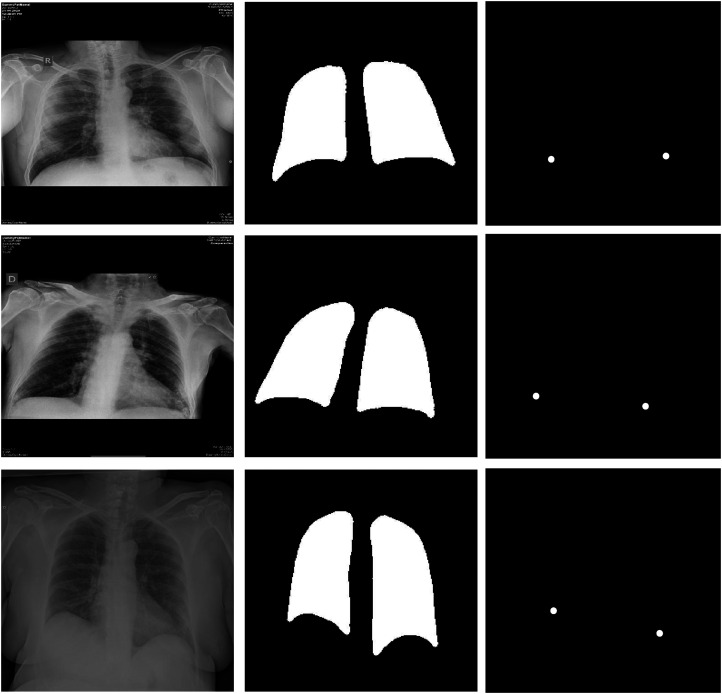
Examples of the CHUAC dataset. First column: original images. Second column: ground truth of the lung regions. Third column: ground truth of the location of the hemidiaphragms’ points.

The current study was approved by the corresponding ethics committee with the code 2020-007. In order to comply with the ethics requirements, all the patients were conveniently anonymized before being sent to any external collaborator. Moreover, all the images were securely stored in appropriate private servers that restricted the access to only the members of the project. All the processes were performed following a protocol agreement with the hospital board. It is important to note that all the cases were visually inspected by the CHUAC staff, to find evidences of COVID-19 affectation. This visualization was corroborated with an RT-PCR test. Some representative examples of the dataset can be seen in Figure 1.

The used dataset represents a Western countries’ population, more precisely, a subset of the Spanish population located at Galicia. The studied cohort includes a set of patients with a mean age of 65.99±18.04 years old. Regarding the sex of the patients, 43.71% are females and the remaining 56.29% are males. Furthermore, it can be added that the Galician population has very particular traits, closely related with the Portuguese population and even a great north African ancestry, making it notably different to other Spanish regions.^38^ While creating this dataset, we have adhered to strict requirements regarding dataset inclusion. These criteria were carefully designed in collaboration with the institution that supplied the data, to ensure that they accurately represent the population that we pretend to model. Moreover, the mentioned criteria have taken into account aspects related with biases that could appear. Particularly, Álvarez-Rodríguez et al.^39^ suggested that the variable sex have no significant influence on COVID-19 involvement, while slight variations could be evidenced between sexes in certain age groups according to Statsenko et al.^40^. Alongside that, both works agree that the age is a significant variable and must be taken into account regarding its possible impact on model performance and the way the results should be discussed. All these elements were considered in the previously mentioned criteria.

To end this section, it is remarkable that, despite using a dataset of portable chest X-ray images (and, therefore, with a lower quality, level of detail and usually with a notable presence of artifacts) we have developed this methodology given that previous state-of-the-art works have demonstrated a great capability handling this kind of input despite the mentioned issues.^41,42^

### Software and hardware resources

The implementation of the methodology herein presented was done using Python 3 (Version 3.8.10). Firstly, for this implementation, it was necessary to use several libraries that are described in Table 1. The main framework chosen for this work was the library torch alongside torchvision, that enables to train and validate computer vision systems using deep learning models. Both libraries were configured with CUDA support, allowing to speed up the training and inference processes with hardware acceleration. Moreover, it was also necessary to add some functionalities from other computer vision and imaging libraries: opencv and scikit-image. In the same line, other functionalities were required from scikit-learn, a machine learning library, to obtain the evaluation metrics. Furthermore, the library pandas was used to work with CSV files and numpy enabled to work with arrays in Python. Secondly, we also specify the characteristics of the used hardware in Table 2. In particular, the experimentation of this work was performed using an NVIDIA Tesla A100 with 2 GPUs of 80 GB each and the driver Version 460.106.00.

**Table 1.** Software libraries and versions used to implement the methodology presented in this work.

Name	Version	Description
grad-cam	1.4.8	Library to visualize the activation maps of the models.
matplotlib	3.6.1	Library used for the graphical visualization of the data.
numpy	1.23.4	Numpy enables the use of arrays in Python.
opencv	4.6.0.66	OpenCV is used to perform computer vision tasks.
pandas	1.5.1	Library that enables the data analysis.
torch	1.12.1+cu116	Library to work with deep learning models.
torchvision	0.13.1+cu116	This library adds additional features to torch library.
scikit-image	0.19.3	This library includes functions to work with images.
scikit-learn	1.1.2	Scikit-Learn enables the work with machine learning models.
scipy	1.9.1	Scipy was used to perform statistical tests

**Table 2.** Hardware resources that were used to execute the implementation of the methodology.

Name	Description
OS	Ubuntu 20.04.5 LTS (Focal Fossa)
Kernel	5.4.0-131-generic
Architecture	x86-64
CPU	AMD EPYC 7763 64-Core Processor
RAM	503.9 GiB
Hard Disk	1007 GB

### Methodology

**Figure 2.**
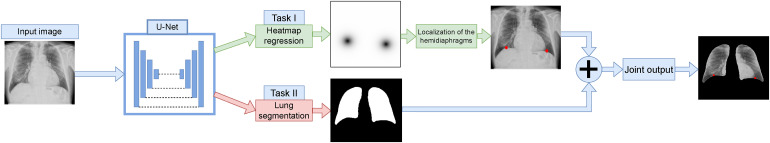
Description of the different tasks performed in the methodology, being heatmap regression task I (that is then used for the detection of the points of the hemidiaphragms) and precise lung segmentation task II.

In this work, we propose the novel multi-task paradigm that is depicted in Figure 2. This paradigm simultaneously performs task I of heatmap regression (from which the landmarks of the hemidiaphragms are later localized) and task II of precise lung segmentation. For this methodology, it is necessary to adapt the network architecture and propose a loss function for each task. All these aspects are deeply detailed in this section.

*Heatmap regression (localization of the landmarks of the hemidiaphragms).* Given the coordinates of an arbitrary landmark, the heatmap is computed as follows. Initially, it is necessary to compute the distance between the coordinates of each pixel in the image with the coordinates of the target landmark. To compute the distance, several metrics such as Minkowski^43^, Mahalanonbis^44^ or cosine similarity distance^45^ can be used. In particular, for the methodology herein proposed, we have adapted the paradigm proposed by Hervella et al.^35^, as we considered that it was the most closely related to our proposal. This paradigm contemplates the use of the Euclidean distance, a commonly employed approach in regression problems, as it is a recognized method in the state-of-the-art both for medical imaging and other domains^46–48^. Consequently, the heatmap will be obtained computing the Euclidean distance between each point of the image and the target. The Euclidean distance is calculated as expressed in equation (1):

(1) $$d(x_{i},y_{i})=\sqrt{(x_{i}-x_{T}{)}^{2}+(y_{i}-y_{T}{)}^{2}}$$

where the pair $\left(x_{i},y_{i}\right)$ denotes the coordinates of an arbitrary point of the image and the pair $\left(x_{T},y_{T}\right)$ denotes the coordinates of the target landmark.

**Figure 3.**
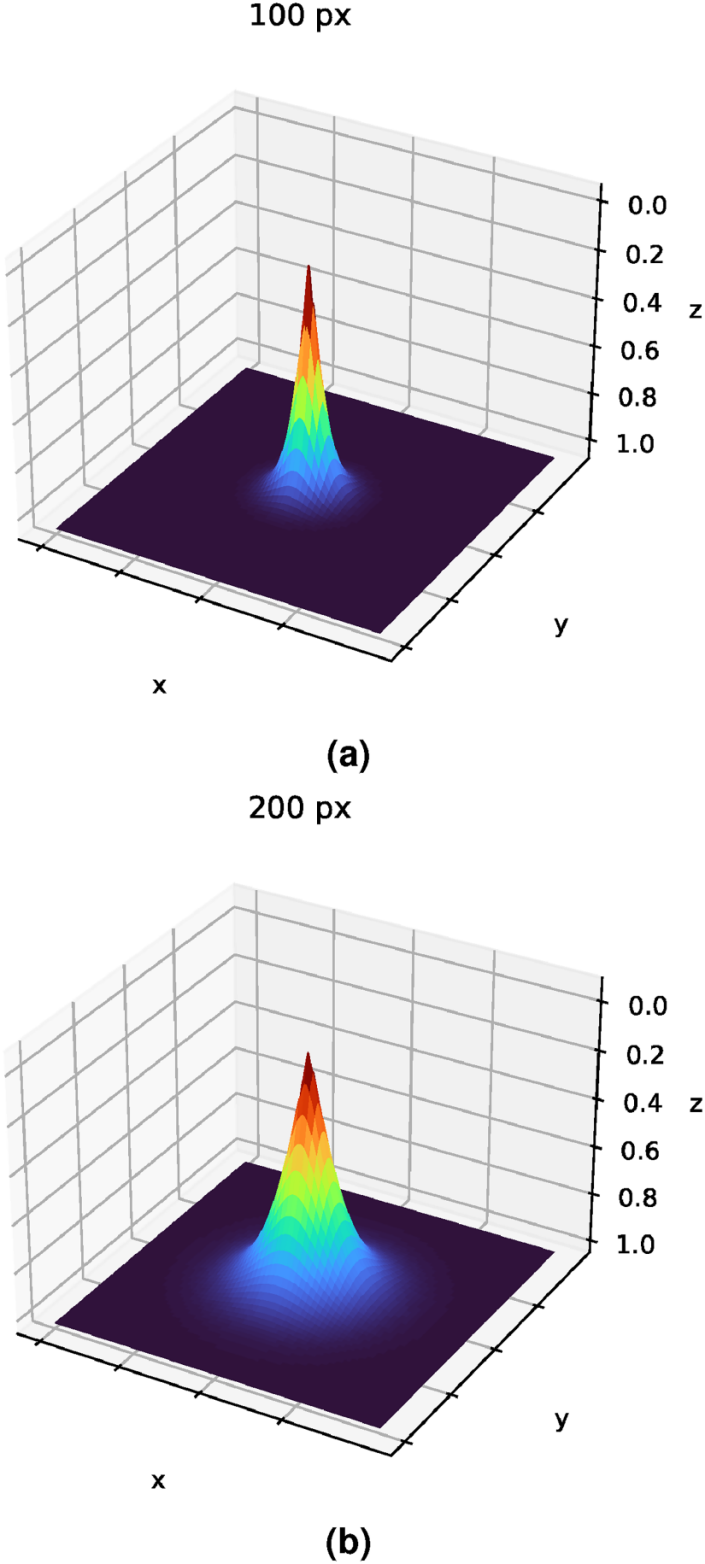
Examples of how the saturation distance β correlates with the heatmap regression output. (a) β= 100 px and (b) β= 200 px.

Nevertheless, the issue of using the expression of the Euclidean distance is that it can give an excessive importance to the distant pixels, an aspect that could lead the model to have a worse performance. To avoid this effect, an exponential decay is applied. In this way, the closest pixels will be given a great importance, while this value will saturate for the most distant pixels. Then, the heatmap will be calculated following the formula stated in equation (2):

(2) $$h(x,y)=\tanh\;(d(x,y)\frac{\pi }{\beta })$$

where h(x,y) refers to the pixel value of the heatmap H at position (x,y) , tanh to the hyperbolic tangent function, and β is the saturation distance. Figure 3 shows how the saturation distance changes the output of the heatmap regression for an arbitrary point, obtaining that this parameter basically defines the radius of the produced heatmap.

Finally, the model will be trained comparing the ground truth with the predicted output using the expression of the MSE loss. This predicted output is an image with the same resolution as the input, given the architectural design that is being used, where the intensity value of each pixel represents its probability of being the actual target point.

Therefore, denoting the loss of the obtained heatmap regression to localize the hemidiaphragms’ landmarks as $L_{hem}$ , the expression is stated in equation (3):

(3) $$L_{hem}=\frac{1}{N}\sum ({\hat{H}}_{i}-H_{i}{)}^{2}$$

where ${\hat{H}}_{i}$ is an arbitrary heatmap predicted by the model, $H_{i}$ its corresponding ground truth, and N is the total number of samples.

*Lung segmentation.* The segmentation loss, denoted as $L_{segm}$ was calculated using the expression of the Dice loss, that is, expressed in equation (4):

(4) $$L_{segm}=1-2\frac{\left|\hat{S}\cap S\right|}{\left|\hat{S}|+|S\right|}$$

where $\hat{S}$ refers to the segmentation predicted by the model and S is the segmentation ground truth.

For the multi-task learning, it is necessary to define a joint expression that merges the losses of the two proposed tasks. The main aim of this expression is to balance the importance that is given to each task. This balance becomes more important due to the fact that the loss of each task can range in a different magnitude. For that reason, two weight values must be defined, $\lambda_{hem}$ and $\lambda_{segm}$ for the heatmap regression loss and the segmentation loss, respectively. Then, the formula of the joint loss is expressed as stated in equation (5):

(5) $$L_{\;joint}=\lambda_{hem}*L_{hem}+\lambda_{segm}*L_{segm}$$

### Network architecture and training details

**Figure 4.**
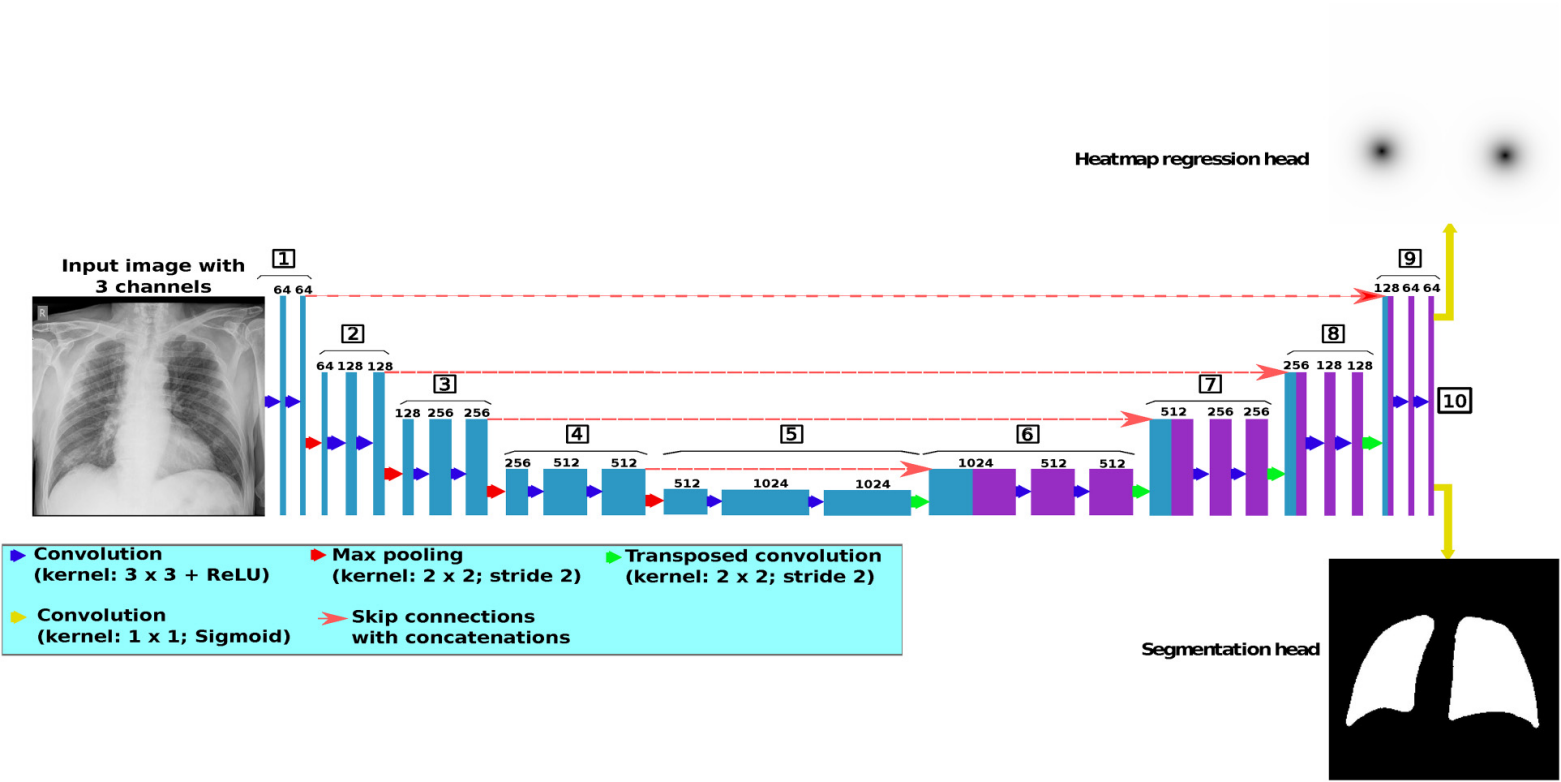
Diagram of the U-Net architecture, adapted for the multi-task paradigm proposed in this work. This architecture is composed of 10 different parts, with four downsampling blocks (encoder), four upsampling blocks (decoder), a block that joins the encoder with the decoder and a final 10^th^ block with a head for each performed task.

Regarding the used network architecture, we adapt the original U-Net structure^49^ given its suitability for medical imaging tasks. This architecture is detailed in Figure 4. The U-Net has an encoder–decoder structure. The encoder part has four downsampling blocks (blocks 1–4 in the diagram), while the decoder is composed of four upsampling blocks (blocks 6–9 in the diagram). The aim of block 5 is to join both parts. Each downsampling block has two convolutional layers (with a kernel of size 3×3 followed by a ReLU activation function) and a max pooling layer (with a kernel of 2×2 and a stride with size 2). Regarding the decoder part of the network, each upsampling block is composed of three layers, with two convolutional layers (having the same characteristics as in the case of the encoder part) and one transposed convolution layer (with a kernel of 2×2 and a stride of size 2). For the purposes of this work, it is necessary to adapt the U-Net architecture to a multi-task paradigm. Therefore, we add two different heads at the output of the network, one for the heatmap regression and another for the precise lung segmentation.

The pipeline of the training process for each task was inspired in previous similar works^50–52^. In particular, it was performed during 200 epochs, with a learning rate of $\alpha =10^{-5}$ optimizing with the ADAM algorithm^53^. The dataset was split using a random holdout with the 60% of the samples for training (setting up a mini-batch size of 1), 20% of the samples for validation, and 20% of the samples for test. As data augmentation, we perform brightness and contrast changes with a probability of 50%, setting up a factor that is randomly selected from range [0.5, 1.5]. Moreover, we also apply a random rotation between $-30^{\circ }$ and $3^{\circ }$ with a probability of 50% and a horizontal flip with a probability of 25%. These transformations are all applied on both input and target images, except in the case of the brightness and contrast changes, given that applying this type of transformation on output images is irrelevant for the learning process of the model. Finally, it is important to remark that the training process is repeated five times with a different random splitting each time, to understand the global behavior of the model, so the results can be reported with the mean and the standard deviation values. This enables to demonstrate the stability and robustness of the proposal in the different splits.

Finally, in order to provide explainability in our study, we have also included a qualitative evaluation of the model with the activation maps of the model. More precisely, we have considered the gradient-weighted class activation mapping algorithm (Grad-CAM or GradCAM)^54^. Furthermore, it is important to note that, in this study, we report the activation map obtained at the output of the fourth encoder blocks.

### Evaluation metrics

To analyze the capabilities of the trained models, we use the metrics that are usually considered in the state-of-the-art. Given that the nature of the two performed tasks is different, the considered evaluation metrics will be particular for each case. With regard to task I, the localization of the landmarks of the hemidiaphragms is evaluated using a similar approach as the one proposed by Marin et al.^37^. Particularly, the accuracy will be measured as the number of points that fall below a threshold divided by the total number of points. To make a more exhaustive analysis, this threshold will be progressive.

In the case of lung segmentation, we considered the same metrics as by Vidal et al.^22^: area under the ROC curve (AUC-ROC), accuracy, precision, recall (heavily used metrics in previous biomedical studies^55,56^), Dice coefficient, and Jaccard index. Denoting TP as the true positives, TN as the true negatives, FP as the false positives, FN as the false negatives, $\hat{S}$ as the segmentation ground truth, and S as the output of the segmentation model, the previously-mentioned metrics are defined as follows:

(6) $$Accuracy=\frac{TP+TN}{TP+TN+FP+FN}$$

(7) $$Dice=2\times \frac{\sum (S\times \hat{S})}{\sum S+\sum \hat{S}}$$

(8) $$Jaccard=\frac{\sum (S\times \hat{S})}{\left(\sum S+\sum \hat{S})-\sum (S\times \hat{S}\right)}$$

(9) $$Precision=\frac{TP}{TP+FP}$$

(10) $$Recall=\frac{TP}{TP+FN}$$

The AUC-ROC is an exhaustive measure that assess the evaluation of the global performance of a model using different operation points^57^.

## Results and discussion

In this section, we present the obtained results of the different designed experimentation. In particular, we performed four different analyses. The first analysis aims to find the most satisfactory saturation distance value ( β ) for the heatmap regression process, being detailed in the “Analysis I” subsection, including a statistical test to find significant differences among the results. The purpose of the second analysis is to find the most appropriate balance between the task of heatmap regression and the task of precise lung segmentation regarding the multi-task paradigm, being described in the “Analysis II” subsection. In the case of the third analysis, the target is to compare the performance obtained on each task when conducted individually and when they are complemented with the other one, being deeply explained in the “Analysis III” subsection. Finally, the aim of the fourth analysis, explained in the “Analysis IV” subsection, is to show and discuss some of the most representative results under a qualitative point of view, including an analysis of the activation maps provided by the network. To end, the “Comparison with the state-of-the-art” subsection provides a discussion comparing the results herein reported with other closely related state-of-the-art works while the “Limitations of the study” subsection explains the main limitations that we have found in our work.

### Analysis I: Ablation study to find the most satisfactory saturation distance value ( β )

In this analysis, we study the impact of the saturation distance ( β ) on the performance of the heatmap regression task. This parameter has been thoroughly studied, with 10 different values: β=1 px, β=10 px, β=25 px, β=50 px, β=75 px, β=100 px, β=125 px, β=150 px, β=175 px, and β=200 px. Moreover, it is necessary to define the thresholds to compute the accuracy as discussed in the “Evaluation metrics” subsection, that in this work have been set to *R*, *R*/2, *R*/5, and *R*/10, where *R* corresponds with 100 px (which is approximately half of the mean width of the lungs). The results obtained for this analysis are detailed in Table 3, showing the individual performances of each lung and a combination between both lungs. Moreover, this table also shows the performance given the four considered thresholds. Globally, as expected, the performance of the model is lower when considering a more restrictive threshold to compute the accuracy. These accuracy values are notably low for β=1 px, with a combined mean accuracy of 34.40% ± 8.13 for threshold *R*/10, 48.88% ± 11.08% for threshold *R*/5, 58.28% ± 11.89% for threshold *R*/2 and 63.88% ± 11.97% for threshold *R*. As a general conclusion from this first β of the ablation study, the model is unable to detect the points accurately in more than half of the cases given the two most restrictive thresholds and unable to detect at least the 70% of the points with the two less restrictive thresholds. These poor results are caused by the small extension of the generated heatmap, that is exclusively restricted to the target landmarks themselves, making it difficult to exploit all the strengths of a fully convolutional approach. Additionally, it is interesting to note that the accuracy is notably lower in the case of the left lung, an aspect that compromises the combined mean accuracy value. This is directly related with the specific characteristics of the human anatomy, as the heart is placed towards the left side of the body, making the delimitation of the left lung more difficult.

**Table 3.** Results of the analysis I, showing the performance of the localization of the hemidiaphragms’ landmarks given the saturation distance β of the heatmap regression, considering different thresholds (*R* = 100). The highest performing configuration for each threshold is highlighted in bold.

		Accuracy (%)			
β (px)	Lung	*R*	*R*/2	*R*/5	*R*/10
1	Right	68.51 ± 13.14	64.18 ± 13.31	55.37 ± 12.89	39.70 ± 9.64
	Left	59.25 ± 10.80	52.39 ± 10.46	42.39 ± 9.27	29.10 ± 6.62
	Both	63.88 ± 11.97	58.28 ± 11.89	48.88 ± 11.08	34.40 ± 8.13
10	Right	99.25 ± 0.82	98.96 ± 0.60	94.18 ± 2.08	81.34 ± 3.27
	Left	96.12 ± 1.66	92.24 ± 2.14	86.87 ± 1.80	68.06 ± 0.56
	Both	97.69 ± 1.24	95.60 ± 1.37	90.52 ± 1.94	74.70 ± 1.92
25	Right	99.40 ± 0.56	98.96 ± 0.60	94.63 ± 0.87	84.33 ± 2.36
	Left	97.91 ± 0.99	95.67 ± 2.47	90.30 ± 2.79	74.48 ± 2.47
	Both	98.66 ± 0.78	97.31 ± 1.53	92.46 ± 1.83	79.40 ± 2.41
50	Right	99.85 ± 0.30	99.70 ± 0.37	95.67 ± 0.87	85.52 ± 2.14
	Left	98.81 ± 1.01	97.76 ± 1.25	92.09 ± 1.01	76.87 ± 1.57
	Both	99.33 ± 0.66	98.73 ± 0.81	93.88 ± 0.94	81.19 ± 1.85
75	Right	100.00 ± 0.00	99.85 ± 0.30	95.82 ± 1.30	84.48 ± 1.45
	Left	99.40 ± 0.56	98.36 ± 0.56	94.48 ± 1.01	78.21 ± 3.28
	Both	99.70 ± 0.28	99.10 ± 0.43	95.15 ± 1.16	81.34 ± 2.37
100	Right	100.00 ± 0.00	99.85 ± 0.30	96.57 ± 1.21	85.97 ± 2.23
	Left	99.25 ± 0.47	98.81 ± 0.37	94.63 ± 3.11	78.96 ± 3.38
	Both	99.63 ± 0.24	99.33 ± 0.34	95.60 ± 2.16	82.46 ± 2.81
125	Right	100.00 ± 0.00	100.00 ± 0.00	96.57 ± 1.54	87.61 ± 1.92
	Left	99.70 ± 0.37	99.10 ± 0.56	94.78 ± 2.06	80.00 ± 3.64
	Both	99.85 ± 0.19	**99.55 ± 0.28**	95.67 ± 1.80	**83.81 ± 2.78**
150	Right	99.85 ± 0.30	99.70 ± 0.37	96.72 ± 1.01	85.97 ± 1.45
	Left	99.55 ± 0.37	99.10 ± 0.73	95.37 ± 1.45	80.15 ± 3.19
	Both	99.70 ± 0.34	99.40 ± 0.55	96.04 ± 1.23	83.06 ± 3.19
175	Right	100.00 ± 0.00	99.85 ± 0.30	97.01 ± 0.67	86.42 ± 1.45
	Left	99.55 ± 0.37	99.10 ± 0.56	94.63 ± 2.56	79.70 ± 2.23
	Both	99.78 ± 0.19	99.48 ± 0.43	95.83 ± 1.62	83.06 ± 1.84
200	Right	100.00 ± 0.00	99.85 ± 0.30	96.87 ± 0.73	86.12 ± 1.92
	Left	99.85 ± 0.30	99.10 ± 0.73	95.97 ± 1.92	78.66 ± 4.18
	Both	**99.93 ± 0.15**	99.48 ± 0.52	**96.42 ± 1.33**	82.39 ± 3.05

From β=1 px onward, there is a considerable improvement of the combined accuracy, with a 74.70% ± 1.92% and a 90.52% ± 1.94% in the case of β=10 px given the most restrictive thresholds. Moreover, for the two less restrictive thresholds, the combined mean accuracy improves in more than 30%, with a 95.60% ± 1.37% and a 97.69% ± 1.24%, respectively. This improvement can also be seen in both lungs individually. In comparison with β=1 px, the accuracy of the left lung is also lower than the accuracy of the right lung. Given the saturation distance β=25 px, the localization of the points is more accurate, with a combined mean accuracy improvement of 4.70% for the most restrictive threshold. For *R*/5, there is also an improvement of 1.94%, 1.71% for *R*/2 and 0.97% for *R*.

The model keeps improving for β=50 px, with an accuracy raise of 1.79% for the most restrictive threshold, 1.42% for *R*/5, 1.42% for *R*/2 and 0.67% for *R* in comparison with β=25 px. From this value of β onward, some improvement can still be seen in some thresholds, but others starts to stall. In particular, for β=75 px, the most restrictive threshold shows a combined mean accuracy of 81.34% ± 2.37% (an improvement of 0.15% in comparison with β=50 px) and of 95.15% ± 1.16% given the threshold *R*/5 (an improvement of 1.27% in comparison with β=50 px). In addition, the threshold *R*/2 shows a 99.10% ± 0.43% while the threshold *R* shows a 99.70% ± 0.28%. This means an improvement of 0.37% in comparison with their respective threshold when β=50 px. From β=75 px, the changes are residual in almost all cases, showing that this value of β is appropriate to detect the points accurately given the three less restrictive thresholds, achieving a combined mean accuracy greater than 95.15% in all cases. However, considering the threshold *R*/10, some important changes can still be seen, having a combined mean accuracy of 82.46% ± 2.81% for β=100 px, 83.81% ± 2.78% for β=125 px, 83.06% ± 3.19% for β=150 px, 83.06% ± 1.84% for β=175 px, and 82.39% ± 3.05% for β=200 px. Considering this threshold, the highest performance is achieved with β=125 px. From this value of β onwards, the performance shows a trend of stabilization. Thus, it can be concluded that β=125 px is enough to capture the whole width of each hemidiaphragm and, therefore, bigger values of β provide no improvements. Considering all these discussed points, and given that the most significant metrics in this problem are the most restrictive, it is concluded that the most appropriate value of β is 125 px.

Overall, the results presented in this analysis demonstrate that the saturation distance β has a great impact on the performance of the heatmap regression. In particular, the smallest values of β have a much lower performance, specially in the case of β=1 px. This occurs because, when β is too low, the model is unable to capture the particular characteristics of the closer regions where the hemidiaphragms are located. As this increases the uncertainty, the model fails to precisely find the landmarks. Moreover, another remarkable conclusion that can be extracted from the results is that the performance of the detection at the left lung tends to be lower than at the right lung. This is probably due to the location of the heart, which is tilted towards the left part of the body, making the lung detection more difficult and, therefore, the left lung detection as well. Other outstanding aspect of the obtained results is that we have obtained a high performance despite using a dataset of images provided by portable devices, with a low quality and level of detail.

Apart from the presented results, this analysis also includes a statistical test. To that end, we have used the method of Wilcoxon signed-rank. This kind of statistical test determines if two series of data follow the same distribution (where *p*-value is >0.05 and, therefore, the null hypothesis is accepted) or if it exists a statistically significant difference (where *p*-value is smaller or equal to 0.05 and, therefore, the null hypothesis is rejected). To evaluate the ablation study presented in this analysis, the experiments have been grouped by their value of β , creating a set of arbitrary pairs ( $\beta_{i}$ , $\beta_{\;j}$ ), thus allowing to perform a one versus one comparison for each of those pairs. These results can be seen in Table 4, making three independent comparisons: considering only the distance errors for the left lung, only the distance errors for the right lung and combining the distance errors of both lungs together. It can be obtained that the three smallest values of β (1 px, 10 px, and 25 px) obtain a significantly higher mean distance error when being compared with the rest of approaches (and, therefore, a worse performance). The remaining β values obtain a statistically similar performance among them. Interestingly, when in an arbitrary comparison is met that $\beta_{i}>\beta_{\;j}$ , the performance of $\beta_{i}$ is always higher than $\beta_{\;j}$ or no statistically different. Moreover, except in some particular cases, the statistical evidence shows an agreement for the three comparisons (left lung, right lung, and both lungs). Globally, the statistical evidence supports some of the discussions that were obtained from Table 4. In particular, the smallest β values have a lower performance in comparison with the rest of the cases (especially when considering β = 1 px), while the performance starts to be notably stable around 50 px, when the statistical comparisons show no significant differences in general.

**Table 4.** Results obtained from performing the Wilcoxon signed-rank test comparing the distance errors among the different β values evaluated in the ablation study.

	1 px	10 px	25 px	50 px	75 px	100 px	125 px	150 px	175 px	200 px
1 px	=	( ⇑ , ⇑ ) ⇑	( ⇑ , ⇑ ) ⇑	( ⇑ , ⇑ ) ⇑	( ⇑ , ⇑ ) ⇑	( ⇑ , ⇑ ) ⇑	( ⇑ , ⇑ ) ⇑	( ⇑ , ⇑ ) ⇑	( ⇑ , ⇑ ) ⇑	( ⇑ , ⇑ ) ⇑
10 px	⋅	=	( ≈ , ⇑ ) ⇑	( ⇑ , ⇑ ) ⇑	( ⇑ , ⇑ ) ⇑	( ⇑ , ⇑ ) ⇑	( ⇑ , ⇑ ) ⇑	( ⇑ , ⇑ ) ⇑	( ⇑ , ⇑ ) ⇑	( ⇑ , ⇑ ) ⇑
25 px	⋅	⋅	=	( ⇑ , ⇑ ) ⇑	( ≈ , ⇑ ) ⇑	( ⇑ , ⇑ ) ⇑	( ⇑ , ⇑ ) ⇑	( ≈ , ⇑ ) ⇑	( ≈ , ⇑ ) ⇑	( ⇑ , ⇑ ) ⇑
50 px	⋅	⋅	⋅	=	( ≈ , ≈ ) ≈	( ≈ , ≈ ) ≈	( ≈ , ≈ ) ≈	( ≈ , ⇑ ) ≈	( ≈ , ≈ ) ≈	( ≈ , ≈ ) ≈
75 px	⋅	⋅	⋅	⋅	=	( ≈ , ≈ ) ≈	( ≈ , ≈ ) ≈	( ≈ , ≈ ) ≈	( ≈ , ≈ ) ≈	( ≈ , ≈ ) ≈
100 px	⋅	⋅	⋅	⋅	⋅	=	( ≈ , ≈ ) ≈	( ≈ , ≈ ) ≈	( ≈ , ≈ ) ≈	( ≈ , ≈ ) ≈
125 px	⋅	⋅	⋅	⋅	⋅	⋅	=	( ≈ , ⇑ ) ≈	( ≈ , ≈ ) ≈	( ≈ , ≈ ) ≈
150 px	⋅	⋅	⋅	⋅	⋅	⋅	⋅	=	( ≈ , ≈ ) ≈	( ≈ , ≈ ) ≈
175 px	⋅	⋅	⋅	⋅	⋅	⋅	⋅	⋅	=	( ≈ , ≈ ) ≈
200 px	⋅	⋅	⋅	⋅	⋅	⋅	⋅	⋅	⋅	=

For each point in the matrix, the symbol = indicates the comparisons, where β values are equal (on the main diagonal), while ≈ presents a comparison with no statistically significant difference and ⇑ is used to represent the situations where there is a statistically significant difference, pointing the β value that obtains the best performance (i.e. the one with a smaller distance error). The significant difference is determined by the test with a *p*-value smaller than 0.05. Except the main diagonal, the rest of cells show the results of the test only considering the distance errors for the first lung, only considering the errors for the second lung and considering the distance errors of both lungs together.

### Analysis II: Analysis of the balance between tasks in the multi-task paradigm

Given that the performed tasks are closely related but different, it is critical to balance the contribution that each one brings to the loss. Setting up the weights of the losses is also important as each one could range in a different magnitude. For that reason, we have designed an exhaustive analysis to determine the most balanced configuration. Totally, we have performed eight different experiments, each one with a particular balance of the loss components. In particular, each experiment progressively gives more weight to lung segmentation while giving less importance to the heatmap regression. Denoting $\Lambda_{i}$ as the pair of loss weights ( $\lambda_{hem}$ , $\lambda_{segm}$ ) of an arbitrary experiment i , the configuration of the eight experiments is as follows: Λ1 = (0.100, 0.900), Λ2 = (0.300, 0.700), Λ3 = (0.500, 0.500), Λ4 = (0.700, 0.300), Λ5 = (0.900, 0.100), Λ6 = (0.950, 0.050), Λ7 = (0.975, 0.025), and Λ8 = (0.990, 0.010). To evaluate these experiments, we have used the same criteria as in the previous analysis. This means that a landmark localization will be considered as correct if the distance between the prediction and the ground truth falls below a threshold. Therefore, following the same approach as in the analysis I, the chosen thresholds will be *R*, *R*/2, *R*/5, and *R*/10, where *R* = 100 px. In the case of the precise lung segmentation, we have performed an evaluation of the global performance obtained on each configuration using the Dice score.

Firstly, Table 5 shows the results of the localization of the hemidiaphragms’ landmarks given the different configurations of loss weights. There, it can be seen that, given the two less restrictive thresholds (*R* and *R*/2), the mean combined accuracy is at least 99.03% for all the cases. It is interesting to remark this aspect in the case of $\Lambda_{1}=(0.100,0.900)$ , where the heatmap regression task is given the lowest weight. Given the threshold *R*/10, the performance barely surpasses 60% of combined mean accuracy and, given the threshold *R*/5, this accuracy is the only one of the whole ablation study to fall below 90% (this even occurs for both lungs). However, the performance for *R* and *R*/2 matches the values obtained by the rest of the configurations. This is significant of the contribution that the precise lung segmentation task brings in the multi-task paradigm, as it helps to find the extension of the hemidiaphragms.

**Table 5.** Results of the analysis II regarding the localization of the hemidiaphragms’ landmarks in the multi-task paradigm, showing the performance of this task given different combinations of loss weights. The highest performing configuration for each threshold is highlighted in bold.

Λ (loss weights)			Accuracy (%)			
$\lambda_{hem}$	$\lambda_{segm}$	Lung	*R*	*R*/2	*R*/5	*R*/10
0.990	0.010	Right	100.00 ± 0.00	100.00 ± 0.00	96.72 ± 1.38	85.52 ± 3.70
		Left	99.55 ± 0.60	98.96 ± 0.37	94.78 ± 2.21	77.46 ± 3.48
		Both	99.78 ± 0.30	99.48 ± 0.19	95.75 ± 1.80	81.49 ± 3.59
0.975	0.025	Right	100.00 ± 0.00	99.85 ± 0.30	96.72 ± 0.76	87.01 ± 3.08
		Left	99.70 ± 0.37	99.40 ± 0.56	95.07 ± 2.14	80.00 ± 3.35
		Both	99.85 ± 0.19	99.63 ± 0.43	95.90 ± 1.45	**83.51 ± 3.22**
0.950	0.050	Right	100.00 ± 0.00	100.00 ± 0.00	97.91 ± 1.10	86.87 ± 0.76
		Left	99.55 ± 0.37	99.40 ± 0.30	95.37 ± 1.28	77.76 ± 3.73
		Both	99.78 ± 0.19	**99.70 ± 0.15**	**96.64 ± 1.19**	82.31 ± 2.25
0.900	0.100	Right	100.00 ± 0.00	99.70 ± 0.37	96.72 ± 0.60	86.57 ± 2.50
		Left	99.85 ± 0.30	99.10 ± 0.30	95.67 ± 2.02	78.81 ± 4.54
		Both	**99.93 ± 0.15**	99.40 ± 0.34	96.19 ± 1.31	82.69 ± 3.52
0.700	0.300	Right	100.00 ± 0.00	99.85 ± 0.30	96.57 ± 1.12	83.13 ± 4.83
		Left	99.55 ± 0.37	98.96 ± 0.37	95.52 ± 1.42	76.72 ± 3.18
		Both	99.78 ± 0.19	99.40 ± 0.34	96.04 ± 1.27	79.93 ± 4.01
0.500	0.500	Right	100.00 ± 0.00	100.00 ± 0.00	95.82 ± 1.92	82.69 ± 1.66
		Left	99.85 ± 0.30	99.10 ± 0.30	94.03 ± 2.63	73.28 ± 3.70
		Both	99.93 ± 0.15	99.55 ± 0.15	94.93 ± 2.28	77.99 ± 2.68
0.300	0.700	Right	100.00 ± 0.00	100.00 ± 0.00	95.22 ± 1.01	80.75 ± 4.41
		Left	99.70 ± 0.37	98.96 ± 0.76	93.73 ± 1.92	72.69 ± 4.29
		Both	99.85 ± 0.19	99.48 ± 0.38	94.48 ± 1.47	76.72 ± 4.35
0.100	0.900	Right	100.00 ± 0.00	100.00 ± 0.00	89.70 ± 2.64	63.88 ± 5.91
		Left	99.55 ± 0.37	98.06 ± 0.60	87.16 ± 2.89	57.76 ± 2.61
		Both	99.78 ± 0.19	99.03 ± 0.30	88.43 ± 2.77	60.82 ± 4.26

With regard to the most restrictive thresholds (*R*/10 and *R*/5) the differences in accuracy are more noticeable. The combined mean accuracy improves from 60.82% to 76.72% with $\lambda_{hem}=0.300$ in the case of *R*/10. Moreover, given the threshold *R*/5, this performance improvement means a combined mean accuracy raise from 88.43% to 94.48%. From this configuration onward, the two less restrictive thresholds show a stabilized performance when $\lambda_{hem}$ is increased. Moreover, from $\lambda_{hem}=0.500$ to $\lambda_{hem}=0.700$ , the threshold *R*/5 shows an accuracy raise from 94.93% to 96.04%, with a trend of convergence after that. The greatest change in terms of performance is noticeable with the most restrictive threshold *R*/10. In particular, there is a consistent improvement from $\lambda_{hem}=0.100$ to $\lambda_{hem}=0.900$ , with a 60.82% ± 4.26% in the first case and an 82.69% in the latter case. From there, the accuracy converges, despite obtaining the highest performance at $\lambda_{hem}=0.975$ , with a value of 83.51% ± 3.22%. In this analysis, we choose $\lambda_{hem}=0.950$ and $\lambda_{segm}=0.050$ as the optimal configuration. This selection was done given that this is the only configuration that achieves the highest accuracy given two different thresholds (*R*/2 and *R*/5) while keeping a competitive performance in the other 2. Globally, it can be concluded that giving a much higher weight to the heatmap regression than to the lung segmentation is important for the model to effectively localize the landmarks of the hemidiaphragms.

With regard to the task of precise lung segmentation, the global results of this ablation study in terms of Dice score can be seen in Table 6. Overall, it can be seen that all configurations are appropriate for the lung segmentation task, given that the lowest value of Dice is 0.9665 ± 0.0006, achieved when $\lambda_{segm}=0.010$ . However, a slight trend of improvement can be seen when $\lambda_{segm}$ increases, that keeps considerably stable from $\lambda_{segm}=0.100$ to $\lambda_{segm}=0.900$ . In fact, the most appropriate configuration (i.e. the one that achieves the highest performance overall) is $\lambda_{segm}=0.700$ with a Dice score of 0.9695 ± 0.0010. Therefore, under a global point of view, the performance of the lung segmentation is slightly higher when the weight given to this task is also higher but, nevertheless, the performance of this task is satisfactory, independently of the configuration. This can be influenced by the fact that each loss component ranges in a different magnitude. In particular, the loss of this task has a considerably higher magnitude in comparison with the heatmap regression. In this way, the drop in performance is only noticeable when the weight $\lambda_{segm}$ is notably close to 0. Taking into account the great importance of the hemidiaphragms’ landmarks localization in this work, the configuration Λ=(0.950,0.050) is chosen as the highest performing for the multi-task paradigm, considering that it obtained the greatest combined mean accuracy values for two different thresholds while keeping a competitive performance in the other 2. Globally, similarly as in the previous approach, the obtained performance is promising despite the low quality and level of detail provided by the portable chest X-ray captures.

**Table 6.** Results of the analysis II regarding the task of precise lung segmentation given the loss weights of each task in the multi-task paradigm. The results of the highest performing configuration are highlighted in bold.

Λ (loss weights)		Dice
$\lambda_{hem}$	$\lambda_{segm}$	
0.990	0.010	0.9665 ± 0.0006
0.975	0.025	0.9683 ± 0.0008
0.950	0.050	0.9688 ± 0.0012
0.900	0.100	0.9693 ± 0.0008
0.700	0.300	0.9692 ± 0.0011
0.500	0.500	0.9694 ± 0.0011
**0.300**	**0.700**	**0.9695 ± 0.0010**
0.100	0.900	0.9694 ± 0.0013

### Analysis III: Comparison between the performance of the tasks separately and simultaneously

**Figure 5.**
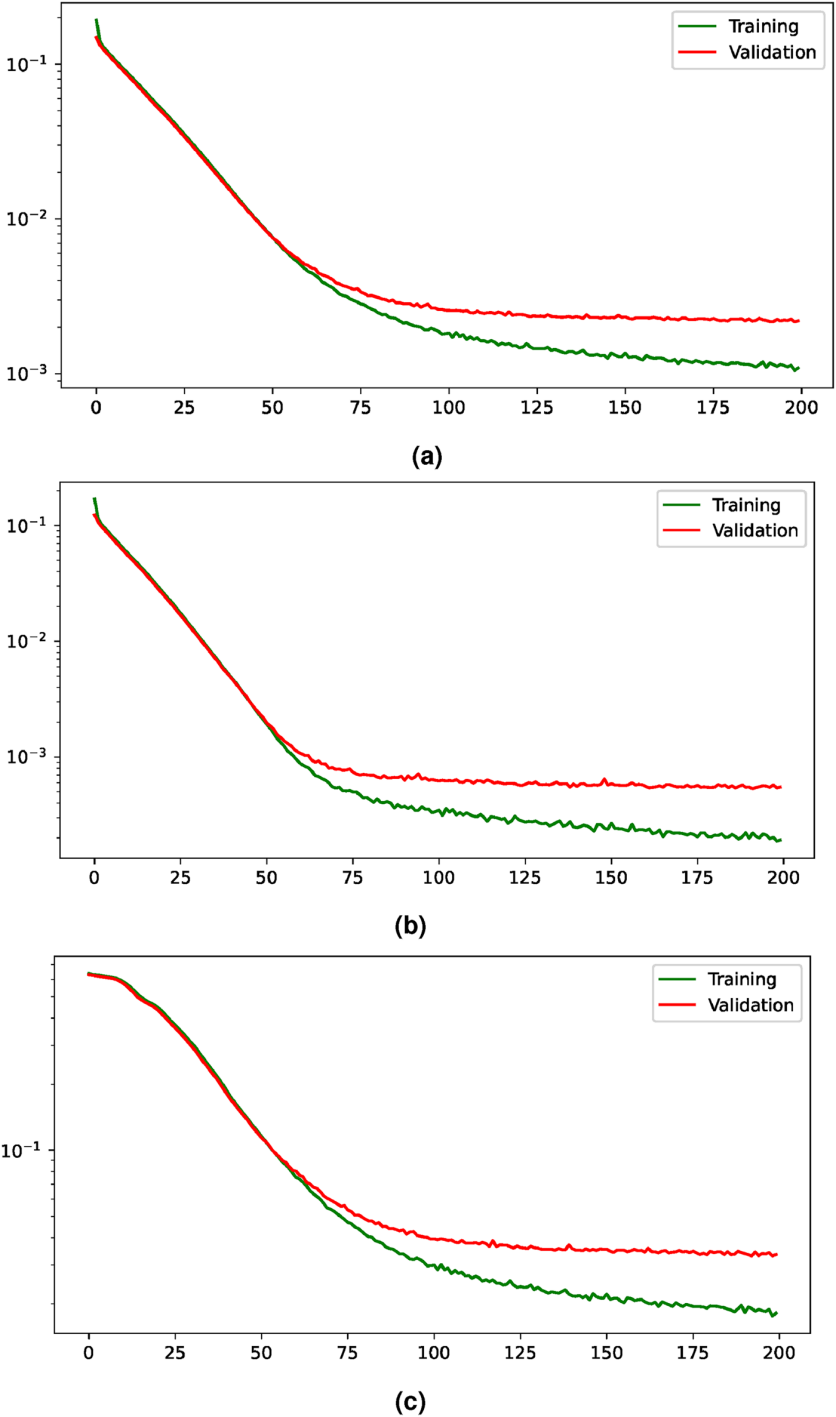
Training and validation losses evolution for the multi-task paradigm. The losses are shown with a logarithmic scale to improve their visualization. (a) Evolution of the joint loss, (b) evolution of the loss for the heatmap regression task, and (c) evolution of the loss for the segmentation task.

For this third analysis, we compare the performance of the tasks (hemidiaphragms’ landmarks localization and precise lung segmentation) when they are performed individually and when they are complemented reciprocally. To do so, we consider the best performing configuration for the heatmap regression task, Λ=(0.950,0.050) . The evaluation of the hemidiaphragms’ landmarks detection will be performed with the same metrics as in the previous analysis (with the thresholds *R*, *R*/2, *R*/5, and *R*/10, where *R* = 100 px) and, in the case of the precise lung segmentation, the comparison will be performed with more detailed metrics (using Jaccard, AUC, accuracy, precision, and recall appart from Dice score). Figure 5 shows the evolution of all the elements that compose the training and validation losses (the loss of the heatmap regression, the loss of the lung segmentation, and the resultant joint loss). The overall conclusion is that all the components achieve convergence in terms of validation loss. The heatmap regression loss achieves convergence around the epoch 75 while the segmentation loss starts to stabilize at approximately the epoch 100. Therefore, the joint loss also converges at around the epoch 100, from where the validation loss keeps stable.

**Figure 6.**
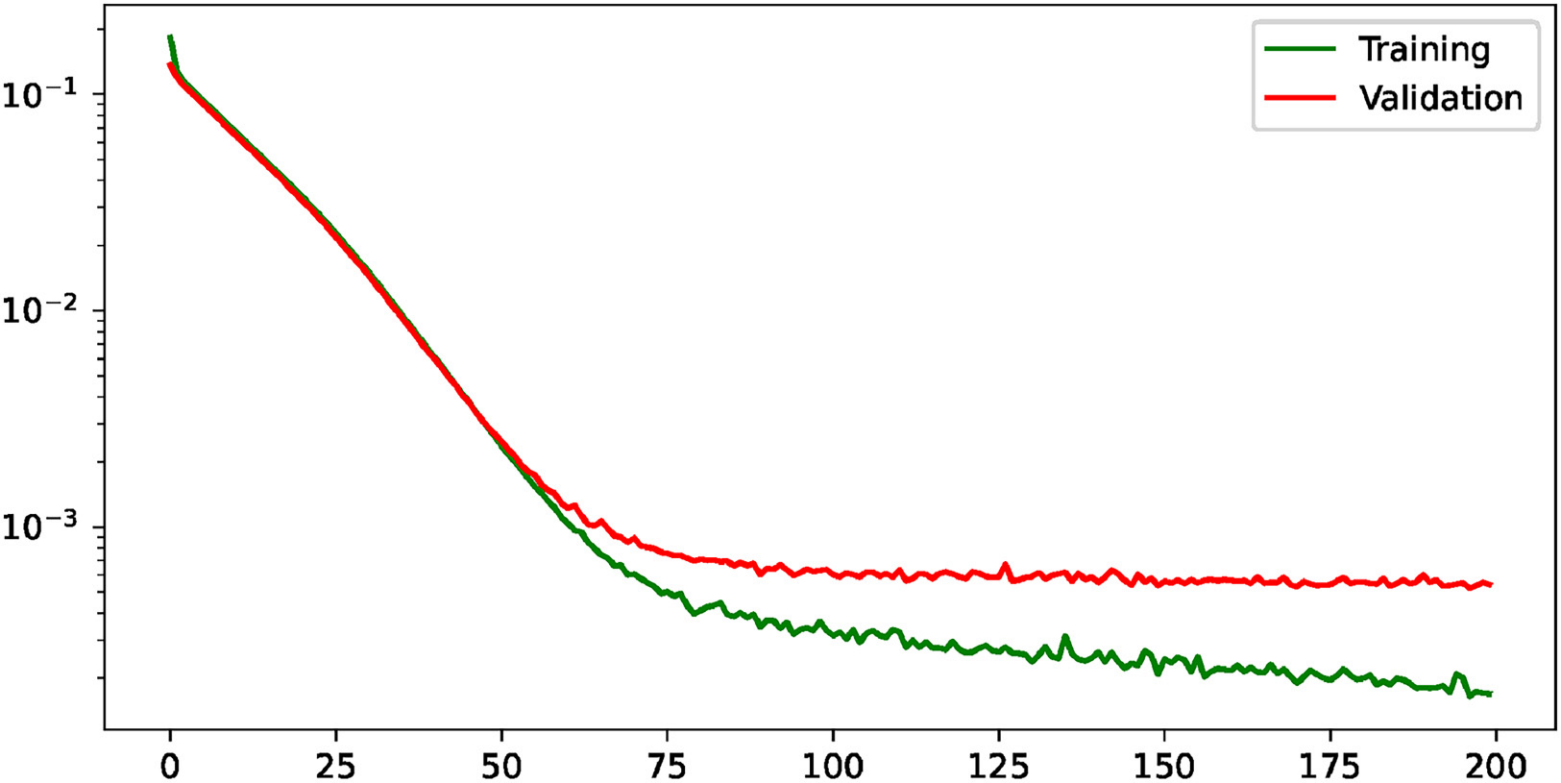
Evolution of the loss during the learning process for the heatmap regression given a saturation distance of β=125 px with the training and the validation subsets, describing a stabilization of the validation loss around the epoch 75. This evolution is shown with a logarithmic scale to improve its visualization.

On the other hand, the evolution of the losses for the heatmap regression when it is performed individually can be seen in Figure 6. This evolution shows an improvement of the training and validation losses until around epoch 75, where the validation starts to stabilize. Moreover, the comparison of the performance obtained when the task is carried out individually and when it is complemented with the precise lung segmentation is shown in Table 7. There, it can be seen that the accuracy values are closely similar. In particular, when the tasks complement each other, the performance is slightly worse in terms of combined mean accuracy given the thresholds *R* and *R*/10. However, there is a slight performance improvement considering the thresholds *R*/2 and *R*/5.

**Table 7.** Comparison of the results obtained when the localization of the hemidiaphragms’ landmarks is performed individually and when it is complemented with the precise lung segmentation given the most appropriate configuration of the balance between tasks. The highest performances for each threshold are highlighted in bold.

	Accuracy (%)			
Lung	*R*	*R*/2	*R*/5	*R*/10
Individually performed				
Right	100.00 ± 0.00	100.00 ± 0.00	96.57 ± 1.54	87.61 ± 1.92
Left	99.70 ± 0.37	99.10 ± 0.56	94.78 ± 2.06	80.00 ± 3.64
Both	**99.85 ± 0.19**	99.55 ± 0.28	95.67 ± 1.80	**83.81 ± 2.78**
Complemented with lung segmentation				
Right	100.00 ± 0.00	100.00 ± 0.00	97.91 ± 1.10	86.87 ± 0.76
Left	99.55 ± 0.37	99.40 ± 0.30	95.37 ± 1.28	77.76 ± 3.73
Both	99.78 ± 0.19	**99.70 ± 0.15**	**96.64 ± 1.19**	82.31 ± 2.25

**Figure 7.**
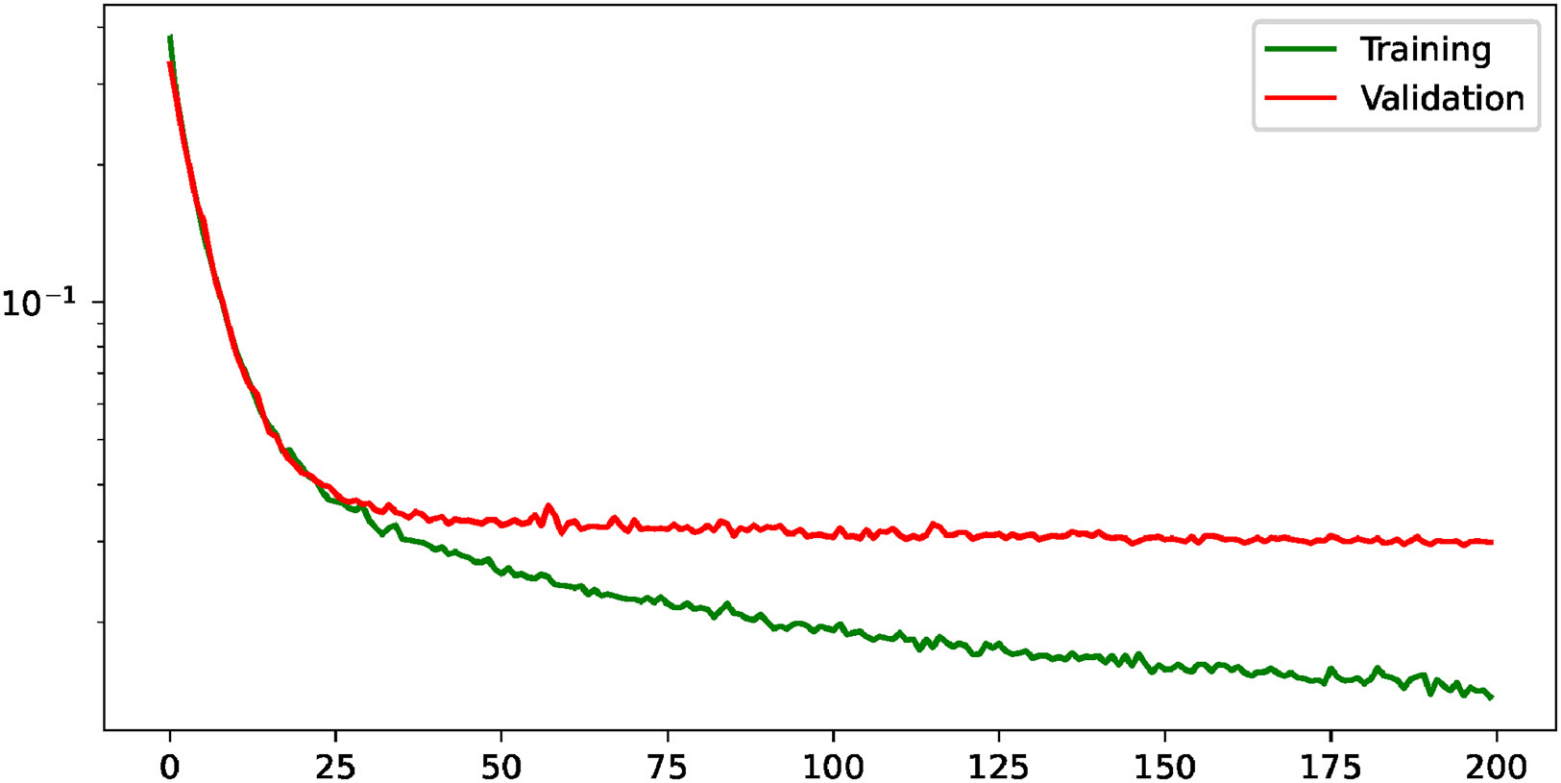
Evolution of the training and validation losses for the task of precise lung segmentation. The values are shown in logarithmic scale to improve their visualization.

With regard to the precise lung segmentation, firstly, the evolution of the training and validation losses can be seen in Figure 7. There, it can be seen that the validation loss keeps improving until it reaches the stability around epoch 50. On the other hand, the comparison of the performance when the task is conducted individually and when it is complemented with the heatmap regression is shown in Table 8. From there, it can be extracted that the multi-task paradigm obtains a competitive performance, with close values in all metrics and a slight improvement in terms of AUC, with a raise from 97.66% ± 0.18% to 98.21% ± 0.18%. Overall, the system is able to accurately perform both tasks despite the particular characteristics of the portable chest X-ray images, in the same line as in the previous analyses.

**Table 8.** Comparison of the results obtained in the task of precise lung segmentation when it is performed individually and when it is complemented with the heatmap regression, using the most suitable configuration of the balance between tasks. The best performing results are highlighted in bold.

Dice (%)	Jaccard (%)	AUC (%)	Accuracy (%)	Precision (%)	Recall (%)
Individually performed					
**96.98 ± 0.06**	**94.21 ± 0.10**	97.66 ± 0.18	**98.35 ± 0.03**	**97.11 ± 0.24**	**96.94 ± 0.25**
Complemented with heatmap regression					
96.88 ± 0.12	94.00 ± 0.22	**98.21 ± 0.18**	98.29 ± 0.08	97.07 ± 0.40	96.76 ± 0.32

### Analysis IV: Qualitative analysis of the obtained results

**Figure 8.**
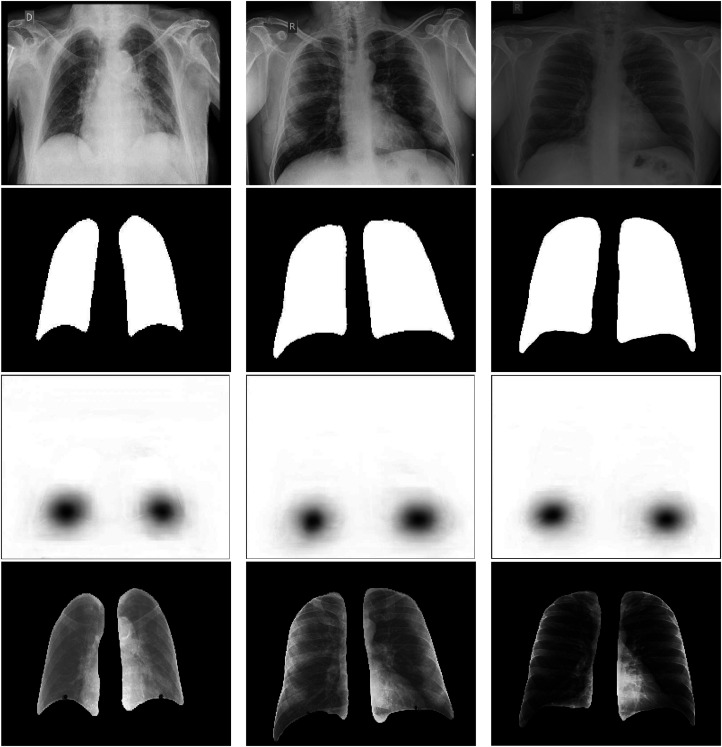
Examples of the multi-task paradigm proposed in this work. Each column represents a particular example. First row: input images that are fed to the model. Second row: precise lung segmentation results. Third row: results of the heatmap regression. Fourth row: simultaneous segmentation and the results of the localization of the hemidiaphragms’ landmarks. The blue point denotes the ground truth and the red point denotes the point predicted by the model.

In this last analysis, we study the obtained results under a qualitative point of view. For this specific analysis, we have selected the best model that was chosen in the analysis III given its suitability for the localization of the hemidiaphragms’ landmarks: Λ=(0.950,0.050) . Some examples of the model output can be seen in Figure 8. This figure depicts the input image and their corresponding outputs: the mask obtained for precise lung segmentation and the heatmap that was regressed from the original input. The binary image corresponding to the lung segmentation shows well-defined boundaries and shapes that correspond with a lung-like structure. Moreover, in the case of the heatmap regression, it can be seen that the output of the model depicts two rounded shapes darker at their center and lighter at their boundaries, placed in the proper position, that is, around the hemidiaphragms that must be localized. Finally, these examples also show the result of executing the method to solve both tasks simultaneously. In those results, the ROIs of the lungs are appropriately defined and the coordinates of the predicted point are precisely close to their corresponding ground truth. From this, it can be concluded that the proposed methodology is able to simultaneously perform both tasks with a high accuracy. In this last analysis, we can extract the same idea as in the previous cases: the system performs the tasks accurately despite the limited level of detail and quality of the portable chest X-ray captures.

**Figure 9.**
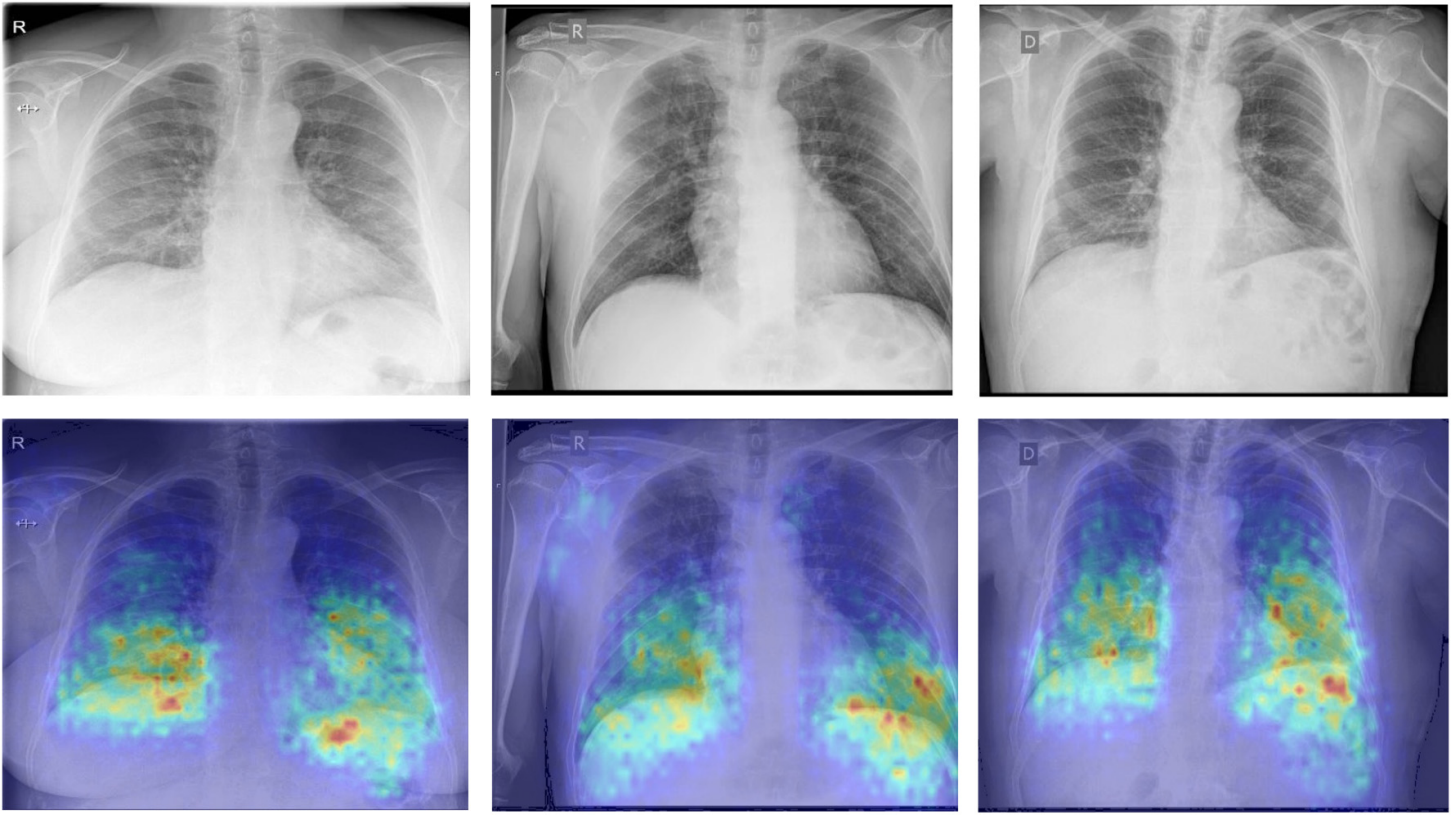
Representative activation maps of input chest X-ray images using GradCAM, where the redder tones represent the activations with a higher intensity and the darker blue tones represent the activations with a smaller intensity.

Regarding the evaluation of the obtained activation maps, some representative examples can be seen in Figure 9. There, it is shown that the model gives a greater activation to those regions of the image located around the lower part of the lungs, with a noticeable (but much lower) level of activation in the contours of the lungs. This defines the main priorities of the model, that gives more importance to locate the points of the hemidiaphragms rather than the contour of the lungs (very relevant for the lung segmentation).

### Comparison with the state-of-the-art

Regarding the comparison with other works, it is important to clarify some important challenges that must be faced. Our contribution provides a unique, fully automatic multi-task approach that simultaneously identifies the location of the hemidiaphragms and achieves a precise lung segmentation. However, despite performing both tasks, the primary innovation of our work resides in the detection of the hemidiaphragms’ landmarks, while the lung segmentation serves as an auxiliary task. Given the pioneering nature of our work, a direct comparison with existing methods presents some important challenges. This is primarily due to the lack of datasets that incorporate manual ground truth labels that fit to the specific tasks that are performed in this work. Nevertheless, a comparison with other previous lung segmentation methods can be performed. In particular, our work achieves a dice score of 0.9688 (complementing the task of lung segmentation with the heatmap regression), while the work of Vidal et al.^22^, that uses portable chest X-ray images from a similar dataset as the one considered in this work, reports a dice score of 0.9447 using. Moreover, other works with different public datasets of images obtained with fixed chest X-ray devices achieve a dice score of 0.9421^21^ and around 0.9500^23^. As it can be seen, the comparison indicates that our results are consistent with the metrics reported by established research, and even slightly higher than other similar approaches. Once again, it is necessary to point out that this comparison has been in different conditions, with different datasets and experimental schemes and, despite trying to make the fairest comparison possible, it must be taken cautiously. On the other side, regarding the localization of the hemidiaphragms’ landmarks, the unique premise of our research means that there are no directly comparable studies in the existing literature.

### Limitations of the study

In this study, there are several areas where we can point out some limitations. Firstly, the used dataset is representative of a very particular demographic group. This makes it necessary to perform small adaptions to the methodology in case that another different dataset to study has very different characteristics from those presented in our study. Secondly, regarding the clinical relevance, despite that this automatic computational methodology was developed together with the clinical professionals to whom it is directed, there are still some points that must be analyzed. This is necessary to ensure that the manual, tedious, time-consuming and error-prone process that the clinicians are currently followed is properly adopted in the daily practice. In the presented work, aspects like the user-friendliness and other elements that could suppose a barrier for adoption, are left undiscussed. Moreover, the potential final clinical applications like the diaphragmatic function quantification or the extraction of clinically relevant biomarkers from lungs are left unexplored in this work. Finally, despite the exploration of different error metrics for the hemidiaphragm points’ localization is interesting, this work has only evaluated the performance obtained with the Euclidean distance, the one that was found to be the naive approach.

## Conclusions

In this work, we have proposed a novel fully automatic methodology to simultaneously predict the location of representative hemidiaphragms’ landmarks and precisely segment the lungs in portable chest X-ray images from COVID-19 patients following a multi-task paradigm. The prediction of the hemidiaphragms’ landmarks location was performed supported by the so-called heatmap regression, a method to predict the likelihood for an arbitrary pixel of the image to be the actual target point. The precise lung segmentation was developed following an end-to-end fashion. For the aims of this work, the U-Net architecture was adapted including two output heads, one corresponding to heatmap regression and another corresponding to precise lung segmentation. To study the suitability of this method, four different analyses were performed. The first analysis aimed to study the most appropriate saturation distance value for the heatmap regression (that directly corresponds with the heatmap extension). The second analysis was conducted to study the most appropriate balance between the two tasks regarding the multi-task paradigm. In the case of the third analysis, we present a comparison between the results obtained in each task individually (i.e. heatmap regression and lung segmentation independently, without the additional contribution of the other task) and the scenario when the tasks complement each other. Finally, in the fourth analysis, we studied the outputs obtained by the model under a qualitative point of view. The results obtained in this work demonstrate the feasibility to localize the landmarks of the hemidiaphragms and the regions of interest of the lungs in chest X-ray images, that can improve the performance of other tasks, such as automatic screening. It is remarkable that this high performance has been obtained despite feeding the system with portable chest X-ray images, that provide a lower quality and level of detail in contrast with fixed machinery and that present a great variability with regard to the position of the patients, given that they can be placed less precisely. To the best of our knowledge, this is the first work that simultaneously performs both the localization of the hemidiaphragms’ landmarks and the precise lung segmentation using a CNN architecture.

The proposed methodology has a great potential in the clinical context, as it could help to perform relevant analyses in the field of COVID-19 and other pulmonary pathologies, given the importance of evaluating the diaphragm and other relevant parts of the lung anatomy such as the parenchymal tissue in some pulmonary diseases to measure the extent of disease damage. Thanks to the methodology, the clinicians could rapidly and accurately be assisted when dealing with great patient populations. In particular, the methodology could be used, among other tasks, to quantify the diaphragmatic function and to extract relevant biomarkers indicative of pathological scenarios. In the same line, the results herein presented, despite being mainly intended to post-COVID-19 studies could also be taken as reference to perform similar studies with other pathologies or even different medical imaging modalities and devices. Other possible area of future work exploration is the evaluation of the performance using alternative distance error metrics different from the Euclidean distance, such as the Minkowski, Mahalanobis, or cosine similarity distance.
